# Inter-rater reliability of the extended Composite Quality Score (CQS-2)

**DOI:** 10.3389/fmed.2023.1201517

**Published:** 2023-08-17

**Authors:** Steffen Mickenautsch, Stefan Rupf, Ivana Miletić, Ulf Tilman Strähle, Richard Sturm, Faheema Kimmie-Dhansay, Kata Vidosusić, Veerasamy Yengopal

**Affiliations:** ^1^Faculty of Dentistry, University of the Western Cape, Bellville, South Africa; ^2^Department of Community Dentistry, Faculty of Health Sciences, School of Oral Health Sciences, University of the Witwatersrand, Johannesburg, South Africa; ^3^Review Centre for Health Science Research, Johannesburg, South Africa; ^4^Synoptic Dentistry, Saarland University, Homburg, Germany; ^5^Department of Endodontics and Restorative Dentistry, School of Dental Medicine, University of Zagreb, Zagreb, Croatia; ^6^Department of Operative, Preventive and Paediatric Dentistry, Charité – Universitätsmedizin Berlin, Berlin, Germany; ^7^Department of Community Oral Health, Faculty of Dentistry, University of the Western Cape, Bellville, South Africa

**Keywords:** Composite Quality Score, clinical rial, trial appraisal, inter-rater reliability, systematic review

## Abstract

**Aim:**

To establish the inter-rater reliability of the Composite Quality Score (CQS-2) and to test the null hypothesis that it did not differ significantly from that of the first CQS version (CQS-1).

**Materials and methods:**

Four independent raters were selected to rate 45 clinical trial reports using CQS-1 and CQS-2. The raters remained unaware of each other’s participation in this study until all rating had been completed. Each rater received only one rating template at a time in a random sequence for CQS-1 and CQS-2 rating. Raters completed each template and sent these back to the principal investigator. Each rater received their next template 2 weeks after submission of the completed previous template. The inter-rater reliabilities for the overall appraisal score of the CQS-1 and the CQS-2 were established by using the Brennan-Prediger coefficient (BPC). The coefficients of both CQS versions were compared by using the two-sample *z*-test. During secondary analysis, the BPCs for every criterion and each corroboration level for both CQS versions were established.

**Results:**

The BPC for the CQS-1 was 0.85 (95% CI: 0.64–1.00) and for the CQS-2 it was 1.00 (95% CI: 0.94–1.00), suggesting a very high inter-rater reliability for both. The difference between the two CQS versions was statistically not significant (*p* = 0.17). The null hypothesis was accepted.

**Conclusion:**

The CQS-2 is still under development, This study shows that it is associated with a very high inter-rater reliability, which did not statistically significantly differ from that of the CQS-1. The promising results of this study warrant further investigation in the applicability of the CQS-2 as an appraisal tool for prospective controlled clinical therapy trials.

## 1. Introduction

The Composite Quality Score (CQS) is a recently established appraisal tool for prospective, controlled, clinical therapy trials based on the deductive falsification approach (1). Trial appraisal that follows such an approach assumes that any trial design characteristic (or the lack thereof) which lies outside a particular set of applied trial appraisal criteria, such as that of the Jadad scale (2) or Cochrane’s Risk of Bias (RoB) tool (3, 4), may completely falsify the truthfulness of trial results. It therefore rejects any confidence in “low bias risk.” Consequently, the approach accepts that, in principle, it is impossible to establish “low bias risk” for any trial. Instead, the CQS follows the concept that, although “low bias risk” cannot be proven, it is possible to establish with high certainty whether bias risk is high. High bias risk is recognized when essential characteristics are absent for a therapy trial to reflect the true effect estimate (5).

The first version of the CQS (CQS-1) was developed as a composite of trial appraisal categories for both systematic and random error. The CQS-1 appeared to have been sufficient for trial appraisal in the field of restorative dentistry, where 681 from the total of 683 trial reports could be rated with high confidence as of high bias risk (6). In addition, Mickenautsch et al. investigated the CQS-1 inter-rater reliability (7). The results showed a very high inter-rater reliability, based on an “almost perfect” strength of inter-rater agreement, according to the Landis/Koch Kappa’s Benchmark Scale (Brennan-Prediger coefficient (BPC) 0.95; 95% CI: 0.87–1.00) (8) that was statistically significantly higher than that of the first version of Cochrane’s RoB tool (7).

However, while the current CQS-1 appeared to have been sufficient for clinical trial appraisal in the field of restorative dentistry, other fields of clinical therapy may contain a higher number of trials that would pass its three simple, non-restrictive criteria. For that reason, a new CQS version (CQS-2) was developed based on meta-epidemiological study evidence. Subsequently, one new criterion concerning double-blinding was added and criteria II and III of the original CQS version were amended (9). These changes raise the questions whether the CQS-2 is associated with a high inter-rater reliability, too, and whether such reliability would statistically significantly differ from that of the CQS-1.

Therefore, this study aimed to establish the inter-rater reliability of the CQS-1 and of the CQS-2 and test the null hypothesis that the inter-rater reliability of the CQS-2 does not differ significantly from that of the CQS-1.

## 2. Methods

The methodology of this study was pre-specified in a protocol, which was made available online prior to the start of the study (10). The final report is given in line with the Guidelines for Reporting Reliability and Agreement Studies (GRRAS) (11).

### 2.1. Rater selection

Each investigator (SM, SR, IM, and VY) selected one independent rater based on the following criteria (to the best of each investigator’s knowledge):

- Knowledge of research methodology;
- Potential and/or demonstrated past interest in conducting systematic reviews of clinical trials;
- Independent from each other and from the investigators (e.g., no joint publication listed in PubMed or other known prior academic collaboration);
- Positive response to the written invitation for participation as rater.

From the potential number of raters contacted, the first raters who agreed to participate were selected. Hence, a total of four independent raters participated in this study. Each investigator (SR, IM, and VY) revealed the identity of their chosen rater to the principal investigator (SM) only and remained unaware of each other’s rater selection until all ratings had been completed.

The number of raters was determined in accordance with a similar study to assess the inter-rater reliability of the CQS-1, published elsewhere (7). Rater selection was quasi-random; that is, although no selection according to a random sequence was conducted, each rater’s acceptance to participate was left to chance. Raters were free to accept or decline a once-off written invitation without any further effort by the investigators to secure study participation.

### 2.2. Rater blinding

In order to assure rater independence, no rater interaction took place during the rating process, thus avoiding any interaction effect on the results. The raters remained unaware of each other’s participation in this study until all rating had been completed. However, in order to investigate the use of the CQS-2 under conditions as close as possible to the practical routine of trial appraisal, the raters were not blinded to the references of the trial reports, the author names and affiliations, nor to acknowledgements and funding sources. In addition, to obtain raters’ informed consent regarding their participation in this study, they received information about the full content of the study protocol. Hence, each rater was aware that their judgment was compared with those of other raters.

### 2.3. Sample size calculation

The number of required trial reports was calculated based on a minimum expected agreement between raters of 70%, and a 95% confidence interval (CI) of 15%, using the appropriate formula for sample size calculation: N = 1/E^2^ (with N = number of required articles and E = confidence interval) (12). In line with the applied sample size calculation method, a minimum number of 44 (rounded to 45) required trial reports were determined.

### 2.4. Trial report selection

All 45 trial reports were selected from PubMed. The references are listed in Supplementary material/Section 1. The database was searched by the principal investigator (SM) using the search term “prospective AND clinical AND controlled AND trial” with the set limits: “Abstract,” “Free full text” [Text availability], “Clinical trial” [Article type], “From 2022/1/1 to 2022/05/31” [Publication date] and “Best match” [Display options]. Citation abstracts were checked whether they described a prospective, clinical, controlled trial, published in the English language. Trials were quasi-randomly selected by choosing the first 45 relevant citations from the resulting search list (trial protocols or trials in publication languages other than English were not included).

### 2.5. Trial rating process

The raters had no extensive expertise in the conduct of systematic reviews of randomized controlled trials. One rater was an epidemiologist and statistician with 8 years’ experience; two were dentists employed in academic institutions with 2–3 years of work experience (one with 2 years’ experience in bias risk assessment), and one was a statistician with 25 years of experience and experience in bias risk assessment but not in the use of trials appraisal tools during systematic reviews.

The rater’s content knowledge of the trials was not assessed. However, due to the quasi-random nature of the trial selection, it was assumed to be slight. No calibration or training in using both CQS versions was carried out. All raters received the study protocol (10) for information about how to apply the CQS-1 and 2 only.

From the principal investigator (SM), each rater received a download link for the 45 trial reports via email and a MS Excel assessment template for both CQS versions was prepared in line with published specifications for each appraisal method (7, 9). Each rater received only one template at a time in a random sequence for CQS-1 and CQS-2 rating. The random sequence (Supplementary material/Section 2) was generated using block randomization (Block size = 2) out of a total of eight rating events. Raters entered their rating results into the template and sent these back to the principal investigator via email. Each rater received their next template 2 weeks after submission of the completed previous template.

### 2.6. The composite quality score

The CQS includes: (i) binary trial report rating per appraisal criterion (Scores: 0 = invalid/falsified, 1 = corroborated); (ii) multiplication of individual rating scores to an overall appraisal score, and (iii) identification of invalid/falsified trial reports based on a zero overall appraisal score.

#### 2.6.1. CQS-1

(a) Systematic error (randomization)

Criterion I: “Randomization” for allocation to treatment groups is in some form reported in the text (Yes = 1/No = 0);

Criterion II: Concealing of the random allocation is in some form reported in the text (Yes = 1/No = 0).

(b) Random error (sample size)

Criterion III: The sample size of any particular treatment group reported in the trial report is not less than N = 200 (Yes = 1/No = 0).

The minimum sample size limit (N) was calculated using the formula: N = {([P1 × (100 − P1)] + [P2 × (100 − P2)])/(P2 − P1)^2^} × f(α,β) (13) and was based on the assumption that the difference in intervention effect between study groups (P1–P2) is not less than 10%, with *α* = 5% and *β* = 20%, that is: f(α,β) = 7.9 (14).

#### 2.6.2. CQS-2

The CQS-2 is an update of the CQS-1 and based on a systematic review with meta-analysis of meta-epidemiological study evidence, concerning the lack of trial design characteristics associated with over- or under-estimation of the correct effect estimate due to systematic error alone (9). In contrast to the CQS-1, the CQS-2 does not include a category for random error. The following criteria were set:

Criterion I: “Randomization” for allocation to treatment groups is in some form reported in the text (Yes = 1/No = 0);

Criterion II:

- Keeping the random allocation sequence in a locked computer file; and
- Translation of the sequence into identical, coded, serially administered containers and/or sealed, opaque envelopes; and
- Reassurance that the person who generated the sequence did not administer it.

are in some form reported in the text (Yes = 1/No = 0);

Criterion III: Double-blinding or the blinding of at least two out of the three groups: trial participants, trial personnel, and trial outcome assessors in some form reported in the text (Yes = 1/No = 0); and.

Criterion IV: The sample size of any particular treatment group reported in the trial is not less than N = 100 (Yes = 1/No = 0).

### 2.7. Statistical analysis

The inter-rater reliabilities for the overall appraisal score of the CQS-1 and the CQS-2 were established by use of the Brennan-Prediger coefficient (BPC) (12). This BPC is given by the ratio (p_a_ − 1/q)/(1–1/q), with p_a_ being the percent agreement and q the number of nominal categories in the rating scale. As in a previous study (7), this study did not use Cohen’s Kappa for inter-rater reliability analysis. Cohen’s Kappa is still the most used agreement measure, mainly due to its correction of agreement expected merely by play of chance. However, it is affected by a paradox that returns biased estimates of the statistic itself (situations where high strength of inter-rater agreement actually produce low values for Kappa). This paradox is generated, because marginal values are not independent from the prevalence of the subject under study and this causes an imbalance in case distribution, resulting in lower kappa values. Hence, Cohen’s Kappa is increasingly being replaced by several newer coefficients, such as the BPC that does not suffer from this shortcoming since it ignores the marginal values (12).

The BPCs of both CQS versions were compared using the two-sample *z*-test. All data analyses were carried out using SAS statistical software (15). A 5% significance level was used.

During secondary analysis, the BPC for each single criterion and each corroboration level for both CQS versions was established. The corroboration levels indicate the number of consecutive criteria a trial has complied with (e.g., level C2 indicates Criterion I and II; level C3 indicates Criterion I, II and III, etc.). After a criterion has been rated with a 0-score, the corroboration level remains the same, even if a following criterion is rated with a 1-score, for example Corroboration level C2: Criterion I and II = 1-score, Criterion III = 0-score, Criterion IV = 1-score (5).

## 3. Results

All four selected raters (FK, KV, RS, and US) completed the rating of all 45 trials with both CQS versions, thus completing a total of 360 evaluations. The rated trials originated from 16 different clinical specialities of which non represented more than 25% of the trials and thus assured a relative even distribution among a variety of medical fields. Most trials were related to surgery (24.4%), followed by internal medicine (15.6%) and dentistry (13.3%). All other 13 clinical specialities contributed less than 10% of the rated trials, each (Table 1). The resulting rating data are presented in Supplementary material/Section 3.

**Table 1:** Characteristics of rated trials.

Clinical specialty	No.	%
Anesthesiology	4	9.0
Cardiology	4	9.0
Clinical immunology	1	2.2
Clinical nutrition	1	2.2
Dentistry	6	13.3
Dermatology	1	2.2
Gynecology	1	2.2
Internal medicine	7	15.6
Neurology	1	2.2
Obstetrics	1	2.2
Oncology	1	2.2
Ophthalmology	3	6.7
Psychotherapy	1	2.2
Reproductive science	1	2.2
Surgery	11	24.4
Urology	1	2.2

The BPC for the CQS-1 was 0.85 (95% CI: 0.64–1.00) and for the CQS-2 it was 1.00 (95% CI: 0.94–1.00). The difference was not statistically significant (*p* = 0.17) and the null hypothesis was accepted. The BPCs for each criterion and each corroboration level are shown in Table 2: For the CQS-1, the BPC for criterion III was the highest (0.86; 95% CI: 0.73–0.99) followed by criterion I (0.71; 95% CI: 0.12–1.00) and criterion II (0.29; 95% CI: 0.00–0.59). For the CQS-2, the highest BPC value was established for criterion I (0.89; 95% CI: 0.70–1.00) followed by criterion IV (0.87; 95% CI: 0.72–1.00), criterion II (0.69; 95% CI: 0.38–1.00) and criterion III (0.54, 95% CI: 0.32–0.76). The results for criterion III of the CQS-1 and criteria I and IV of the CQS-2 reflected an ‘almost perfect’ inter-rater agreement with their upper confidence levels even reaching the maximum value 1.00. The coefficient for the single criteria concerning random allocation, allocation concealment and sample size limit was higher for the CQS-2 than for the CQS-1. However, these differences were not statistically significant (Table 3).

**Table 2:** Brennan-Prediger coefficients with 95% Confidence interval (CI) of the two CQS versions.

	Brennan-Prediger coefficient	95% CI	Strength of inter-rater agreement according to the Landis/Koch Kappa’s Benchmark Scale*
Single criterion/CQS-1			
Criterion I – Random allocation	0.71	0.12–1.00	Substantial
Criterion II – Allocation concealment	0.29	0.00–0.59	Fair
Criterion III – Sample size limit	0.86	0.73–0.99	Almost perfect
Single criterion/CQS-2			
Criterion I – Random allocation	0.89	0.70–1.00	Almost perfect
Criterion II – Allocation concealment	0.69	0.38–1.00	Substantial
Criterion III – Double blinding	0.54	0.32–0.76	Moderate
Criterion IV – Sample size limit	0.87	0.72–1.00	Almost perfect
Corroboration levels/CQS-1			
C1: Criterion I	0.71	0.12–1.00	Substantial
C2: Criterion I + II	0.24	0.00–0.61	Fair
C3: Criterion I + II + III	0.85	0.64–1.00	Almost perfect
Corroboration levels/CQS-2			
C1: Criterion I	0.89	0.70–1.00	Almost perfect
C2: Criterion I + II	0.69	0.38–1.00	Substantial
C3: Criterion I + II + III	0.81	0.57–1.00	Almost perfect
C4: Criterion I + II + III + IV	1.00	0.94–1.00	Almost perfect

*Poor: <0; Slight: 0–0.20; Fair: 0.21–0.40; Moderate: 0.41–0.60; Substantial: 061–0.80; Almost perfect: 0.81–1.00 (8).

**Table 3:** Differences in the Brennan-Prediger coefficients between the components of the different rating tools.

Appraisal category	CQS-1	CQS-2	*p*-value
Random allocation	Criterion I	Criterion I	0.57
Allocation concealment	Criterion II	Criterion II	0.071
Sample size limit	Criterion III	Criterion IV	0.92

The BPC for most corroboration levels suggested “substantial” or “almost perfect” strength of inter-rater agreement for both CQS versions, particularly for the CQS-2; except for level C2 of CQS-1 (BPC 0.24; 95% CI: 0.00–0.61), which indicated “fair” strength of inter-rater agreement only. Notwithstanding, the difference between the coefficient value to that of the CQS-2 was not statistically significant (*p* = 0.069).

## 4. Discussion

### 4.1. Study results

The results of this study show that the CQS-2 is associated with a very high inter-rater reliability (BPC 1.00; 95% CI: 0.94–1.00), which did not statistically significantly differ from that of the CQS-1 (*p* = 0.17). In addition, this study replicated the very high inter-rater reliability for the CQS-1 (BPC 0.85, 95% CI: 0.64–1.00), thus confirming previous results (BPC 0.95, 95% CI: 0.87–1.00) (7).

These results compare favorably to that of previously established inter-rater reliabilities of other evidence appraisal tools: the Jadad scale (BPC 0.70; 95% CI: 0.58–0.82) (7), the Grading of Recommendations, Assessment, Development and Evaluation (GRADE) approach (Intraclass correlation coefficient 0.84; 95% CI: 0.78–0.89) (16) and the second version of Cochrane’s Risk of Bias tool (for overall judgment: Fleiss’s Kappa 0.16; 95% CI: 0.08–0.24) (17).

The results of this study also show that adding one further criterion (the new criterion III) and amending two existing criteria (new criterion II and IV) to the CQS (9) did not negatively affect its inter-rater reliability. The BPC for the added CQS-2 criterion III, regarding double-blinding, was found to be 0.54 (95%CI: 0.32–0.76) only. However, this result still compares favorably to that of previously established results for the bias risk domains “operator blinding” and “evaluator blinding” of the RoB-1 tool [BPC 0.03; 95% CI: −0.22 to 0.28 and 0.27; 95%CI: −0.08 to 0.62, respectively (7)].

It has been observed from previous data (5, 7) that higher corroboration levels were associated with higher Brennan-Prediger coefficient values. The higher the corroboration levels, the more single binary (0/1) scores from single appraisal criteria are multiplied into an overall trial appraisal score. A higher number of multiplied single scores increase the chance of multiplication by a single 0-score, which subsequently would render the overall score as zero. This higher chance of an overall 0-score increases the chance that an independent rater will agree on a 0-score in the overall appraisal of a trial, even when they differ in the rating of a single criterion. Such possible mechanism may explain the consistently very high inter-rater reliability of the CQS. It may also indicate that, in that way, rating errors between individual raters are canceled out and thus a high inter-rater reliability is retained.

However, in this study, a consistent pattern of increasing Brennan-Prediger coefficient per corroboration level was not observed. Both CQS versions showed a decrease in the coefficient at corroboration level C2 (Table 2). Such a decrease may be explained on basis that the coefficient for criterion I was high in both CQS versions and subsequently reduced at C2 level by combination with a lower coefficient for criterion II. The difference between the current results and results from a previous study (7) may have been due to variations in the characteristics of the rated trials. In a previous study by Mickenautsch et al. only trials related to restorative dentistry were rated. Only a small number of these trials reported the application of allocation concealment (CQS-1/criterion II). It thus may have been easier for all raters to agree on a 0-score for this criterion, resulting in a higher Brennan-Prediger coefficient (7). In this study, clinical trials from various medical fields were included instead. In these trials, allocation concealment was more frequently applied but reported in different ways. This may have made trial appraisal more challenging and thus negatively affected the inter-rater reliability.

Notwithstanding such observed differences, the Brennan-Prediger coefficient and its lower confidence limit for the CQS-2 increased steadily from level C2 upwards to corroboration level C4 (i.e., the overall CQS-2 score): BPC C2: 0.69 (95% CI: 0.38–1.00); C3: BPC 0.81 (95% CI: 0.57–1.00) and C4: BPC 1.00 (95% CI: 0.94–1.00) (Table 2).

It was further observed that, although the difference was not statically significant (*p* = 0.071), the Brennan-Prediger coefficient for criterion II for the CQS-2 was higher than that of the CQS-1 (Table 2), despite the former having a more restrictive nature. However, it is possible that, because of the higher restriction level for this criterion, it was easier for raters to agree on a 0-score, thus resulting in a higher Brennan-Prediger coefficient. Our data show that raters agreed far more often on a 0-score for criterion II using the CQS-2 (with no agreement for a 1-score) than with the CQS-1. There was also overall less agreement for both 1- and 0-scores combined when using criterion II with the CQS-1 than with the CQS-2 (Supplementary material).

### 4.2. Study limitations and recommendations for further research

The fact that none of the 45 trials received an overall 1-score by any of the four raters may indicate that a too low sample size may have been calculated. A higher sample size may have resulted in at least a few overall 1-score judgment by some of the raters and, thus, a higher precision of the study results. Further inter-rater reliability studies may include a larger number of trials based on a higher expected agreement percentage than was used in this study (70%). Also, the quasi-random sampling method for trials used in this study caused that a heterogeneous range of different medical fields was included, resulting in an overall slight rater content knowledge only. Usually, raters who participate in a systematic review of trials are experts in the particular field of medicine study and appraise trials of homogeneous content that are related to a specific clinical review question. Such high content knowledge would most likely assist in a higher strength of inter-rater agreement than observed in this study.

The CQS-2 as a trial appraisal tool is still under development. Trials from systematic reviews that have applied the 2nd version of Cochrane’s RoB tool may be re-appraised using the CQS-2 in order to establish whether the direction and magnitude of any pooled effect estimates remain the same. Based on the results of these further investigations, the CQS-2 may be piloted as part of the regular, systematic review methodology for the appraisal of prospective, controlled clinical therapy trials.

## 5. Conclusion

This study shows that the CQS-2 is associated with a very high inter-rater reliability, which did not statistically significantly differ from that of the previous CQS-1. The promising results of this study warrant further investigation into the applicability of the CQS-2 as an appraisal tool for prospective controlled clinical therapy trials in systematic reviews.

## Data availability statement

The original contributions presented in the study are included in the article/Supplementary material, further inquiries can be directed to the corresponding author.

## Ethics statement

Ethical review and approval was not required for the study on human participants in accordance with the local legislation and institutional requirements. Written informed consent from the participants was obtained to take part in this study.

## Author contributions

SM contributed to conception and design of the study and wrote the first draft of the manuscript. US, RS, FK-D, and KV contributed to the investigation. All authors commented, improved the manuscript, read, and approved the final version of the manuscript.

## Conflict of interest

The authors declare that the research was conducted in the absence of any commercial or financial relationships that could be construed as a potential conflict of interest.

## Publisher’s note

All claims expressed in this article are solely those of the authors and do not necessarily represent those of their affiliated organizations, or those of the publisher, the editors and the reviewers. Any product that may be evaluated in this article, or claim that may be made by its manufacturer, is not guaranteed or endorsed by the publisher.
